# Construction of High-Density Genetic Map in Barley through Restriction-Site Associated DNA Sequencing

**DOI:** 10.1371/journal.pone.0133161

**Published:** 2015-07-16

**Authors:** Gaofeng Zhou, Qisen Zhang, Xiao-qi Zhang, Cong Tan, Chengdao Li

**Affiliations:** 1 Department of Agriculture and Food, South Perth, WA, Australia; 2 Australian Export Grains Innovation Centre, South Perth, WA, Australia; 3 Western Australian State Agricultural Biotechnology Centre, Murdoch University, Murdoch, WA, Australia; Nanjing Forestry University, CHINA

## Abstract

Genetic maps in barley are usually constructed from a limited number of molecular markers such as SSR (simple sequence repeat) and DarT (diversity arrays technology). These markers must be first developed before being used for genotyping. Here, we introduce a new strategy based on sequencing progeny of a doubled haploid population from Baudin × AC Metcalfe to construct a genetic map in barley. About 13,547 polymorphic SNP tags with >93% calling rate were selected to construct the genetic map. A total of 12,998 SNP tags were anchored to seven linkage groups which spanned a cumulative 967.6 cM genetic distance. The high-density genetic map can be used for QTL mapping and the assembly of WGS and BAC contigs. The genetic map was evaluated for its effectiveness and efficiency in QTL mapping and candidate gene identification. A major QTL for plant height was mapped at 105.5 cM on chromosome 3H. This QTL with LOD value of 13.01 explained 44.5% of phenotypic variation. This strategy will enable rapid and efficient establishment of high-density genetic maps in other species.

## Introduction

Barley is the fourth most-abundant cereal in the world (http://faostat.fao.org), with uses ranging from food, feed, malting and brewing to being a model organism in molecular research. Genetic maps play a pivotal role in QTL mapping for agronomic traits. Several types of molecular markers have been developed for linkage map construction in barley, with the two most popular being SSR (simple sequence repeat) and DarT (diversity arrays technology). Recently, high-density SNP markers were designed in barley based on cDNA polymorphisms [1]. The downside of these markers is that they must be ready before conducting genetic map construction.

Advances in high-throughput DNA sequencing technology make it possible to construct *de novo* genetic maps. Recently, a number of species have been sequenced. For instance, genomes of two main crops, wheat [2] and barley [3], were sequenced using whole-genome shotgun sequencing technology. Furthermore, genotyping-by-sequencing (GBS), a low cost, reduced representation sequencing method, is becoming a common approach for whole-genome marker profiling in many species. Analysis of a recombination population allows the construction of *de novo* genetic maps. There are limitations, however, to those species with large genome size, such as wheat and barley; so several targeted complex DNA reduction methods have been applied to produce high-quality polymorphism data at a relatively low per sample cost. Yang et al. [4] applied NGS (next-generation sequencing)-based RAD (restriction-site associated DNA)-sequencing technology to construct a lupin genetic map. A total of 94 recombinant inbred lines and their parental lines were sequenced and 8,244 sequence-tagged markers were integrated into linkage groups [4]. In barley, Poland et al. [5] used a novel two-enzyme approach for complexity reduction to genotype bi-parental DH population and anchor over 34,000 SNPs into a reference barley genetic map. The high-density genetic map will facilitate QTL mapping and fine mapping.

Furthermore, high-density genetic maps help in the construction of physical maps, assembly of BAC contigs and whole-genome shotgun sequence contigs. Next-generation whole-genome shotgun sequencing is popular and sequence assembly can be achieved by software, but the difficulty is to link the nearby sequence contigs to each other and provide a linear order of contigs along each chromosome. BAC physical maps, BAC-end sequences and fully sequenced BAC sequences provide a framework for the assembly of barley whole-genome shotgun sequences [3]. However, the development of these resources requires substantial time and labour. Recently, a new method called POPSEQ was introduced to anchor NGS contigs assemblies. Instead of constructing BAC physical maps, high-density genetic maps from population sequencing allow *de novo* production of genetically-anchored linear shotgun sequence contigs [6].

Baudin and AC Metcalfe are the two international benchmark varieties for malting quality from Australia and Canada, which have been used worldwide as parents for commercial barley breeding. They differ in plant height, malting quality and disease resistance. Genetic map construction is the basis for QTL mapping for these traits and eventual identification of underlying genes. The objective of this work was to construct a high-density genetic map using NGS-based RAD-sequencing technology. The high-density markers will facilitate exploration of more genetic markers within QTL regions and conduct fine mapping work. The effectiveness and efficiency of this genetic map was evaluated by mapping QTL for plant height in the population. In addition, some barley BAC contigs (cv. Morex) were anchored to the high-resolution genetic map based on their sequence homology. The genetically linear BAC contigs orders will assist physical map construction in barley.

## Materials and Methods

### Whole-genome shotgun sequencing

Illumina paired-end libraries were generated from genomic DNA of 94 individuals from the AC Metcalfe and Baudin DH population and their parental lines.

RAD sequencing was conducted according to Chutimanitsakun *et al*. [7] with some modifications. The restriction enzyme *Eco*RI was used to replace the restriction enzyme *Sbf*I. Ten single-end sequencing libraries (100 bp) were constructed using eight-nucleotide multiplex identifiers (MID) [8]. Each library consisted of eight test plants. Each plant had a unique MID barcode. The RAD products from these individuals were processed in ten lanes on the NGS platform HiSeq2000. According to their specific eight-nucleotide MID barcodes in each library, the sequencing data were assigned into each of the 96 individual plants [8]. The length of DNA sequences of RAD reads was 100 bp including MID barcodes. After the eight-nucleotide MID barcode sequences were removed, about 92 bp of each RAD read was used in bioinformatics analysis. The RAD reads within each individual plant were first clustered into read tags or contigs based on sequence homology. Namely, RAD reads containing the same DNA sequences within each plant were aligned into one read tag. Clustered tags containing more than 100 RAD reads were removed to avoid detection of SNP markers from repetitive regions [9].

### SNP calling

Sequencing reads were quality trimmed using BWA version 0.6.2 [10]. Duplicate reads were removed using SAMtools [11]. Barley cultivar ‘Morex’ genomic sequence was used as a reference. To identify SNPs in these lines, all pairs of tags were checked for a one or two base pairs difference. SNPs were identified by querying the filtered tags for pairs of sequences with these parameters: identical except for one or two base pairs, present in >80% of the individuals and passed a Fisher Exact test for their independence. Heterozygous calls in an individual line were always discarded. Genotype calls not matching the specified parameters were considered missing values. SNPs with more than 20 missing calls in the population were removed. The genotype was recorded as “A” indicating that it was the same to AC Metcalfe, and “B” for Baudin. Sequence tags with a missing rate less than 7% were chosen for linkage map construction.

### *De novo* genetic map construction

Genetic map construction was performed using MSTMap [12] with the following parameters: population_type DH; distance_function kosambi; cut_off_p_value 0.000001; no_map_dist 25.0; no_map_size 0; missing_threshold 1.00; estimation_before_clustering no; detect_bad_data yes; objective_function COUNT. The resulting map contained six linkage groups. One linkage group with a large gap (above 35 cM) between two markers was subdivided into two groups. The obtained marker orders, orientations and genetic distances were adjusted according to barley seven chromosomes. Barley genome sequences were used to provide some useful information for the arrangement of these high-density genetic maps. The linkage groups were determined by blasting Morex genomic sequences using SNP tag sequences. The genetic maps were drawn in Mapchart 2.2 [13].

### Comparison of genetic maps

The marker sequences were used to blast barley genomic sequences (http://webblast.ipk-gatersleben.de/barley/viroblast.php). The top hits provided the anchored WGS contig ID and POPSEQ genetic positions. All marker genetic positions were compared with Morex and Barke genetic map positions.

### Generation of genetically linear FPC contig order

Marker sequences were blasted Morex genome FPC (finger print contig) sequence database [3]. Markers were anchored to the BAC contigs, chromosome and consensus genetic maps. The genetically linear BAC contigs were re-generated based on the high-density genetic maps.

### Mapping QTL for plant height

The DH population and its parents were planted in the high rainfall agricultural area of Western Australia. Each line was planted in 1 × 5 m plots in a randomized design. Plant height was measured from the average of five individuals in each plot before maturity. The plant average height of each line in all trials was calculated and used for QTL mapping analysis.

The software MapQTL 5.0 [14] was used to conduct QTL analysis for plant height. The files for genotypes, phenotypes and genetic maps were imported into the software. Interval analysis was first performed to estimate the closest markers associated with plant height, and then followed by multiple QTL model (MQM) analysis. LOD threshold values applied to declare the presence of a QTL were estimated by performing whole-genome wide permutation tests in MapQTL 5.0 using 10,000 permutations. The QTL map was generated using Mapchart 2.2 [13].

## Results

### Whole-genome sequencing

Sequences from AC Metcalfe and Baudin mapping populations were generated on Illumina HiSeq 2000 (NCBI accession number: SRP057861). On average, around 15 Gb sequence data were generated per lane. Of the 150 million raw reads per lane, 135 million (90%) were high-quality reads according to the Illuminia filter analysis. The sequence coverage was about 13.5 million reads, and 0.26 fold of the barley genome size for each individual.

Among the 13.5 million sequences, only markers with more than 93% calling rate in the mapping population were selected for linkage map construction. In total, 13,547 markers (25%) with less than 7% missing rate were selected for genetic map construction.

### High-density linkage map

A total of 12,998 were mapped to seven linkage maps, with 549 markers unanchored to any chromosomes. These genetic maps spanned a total length of 967.6 cM with individual chromosomes ranging from 112.2 cM (4H) to 169.4 cM (5H). The number of markers in different chromosomes ranged from 529 on 3H to 4,929 on 7H, which were distributed evenly on each chromosome. On average, the density of markers was around 13 markers per cM. These markers were anchored to a total of 5,147 loci along the seven chromosomes with an average of 2.5 markers at each locus (Table 1). The distance between two nearby markers was unclear based on their genetic distances, thereby we mapped all SNP tags to Morex_contigs. The physical distance between two markers could be estimated.

**Table 1:** SNP markers details across each chromosome.

Chr.	Genetic distance (cM)	Markers	Loci	Markers anchored to BAC contig	Total BAC contig
1H	130.0	560	309	209	79
2H	134.0	2,190	957	734	236
3H	155.0	529	340	199	86
4H	112.2	601	353	192	99
5H	169.4	2,705	1,094	858	293
6H	126.5	1,484	672	463	160
7H	140.5	4,929	1,422	1,499	402
Total	967.6	12,998	5,147	4,154	1,355

We anchored these SNP marker tags to the 6,278 sequenced BACs contigs [3]. A total of 4,154 (~32%) markers were localized to 1,355 existing BAC contigs, with 79 to 402 BAC contigs in each chromosome (Table 1).

### Comparison of genetic maps and physical maps

**Fig 1:**
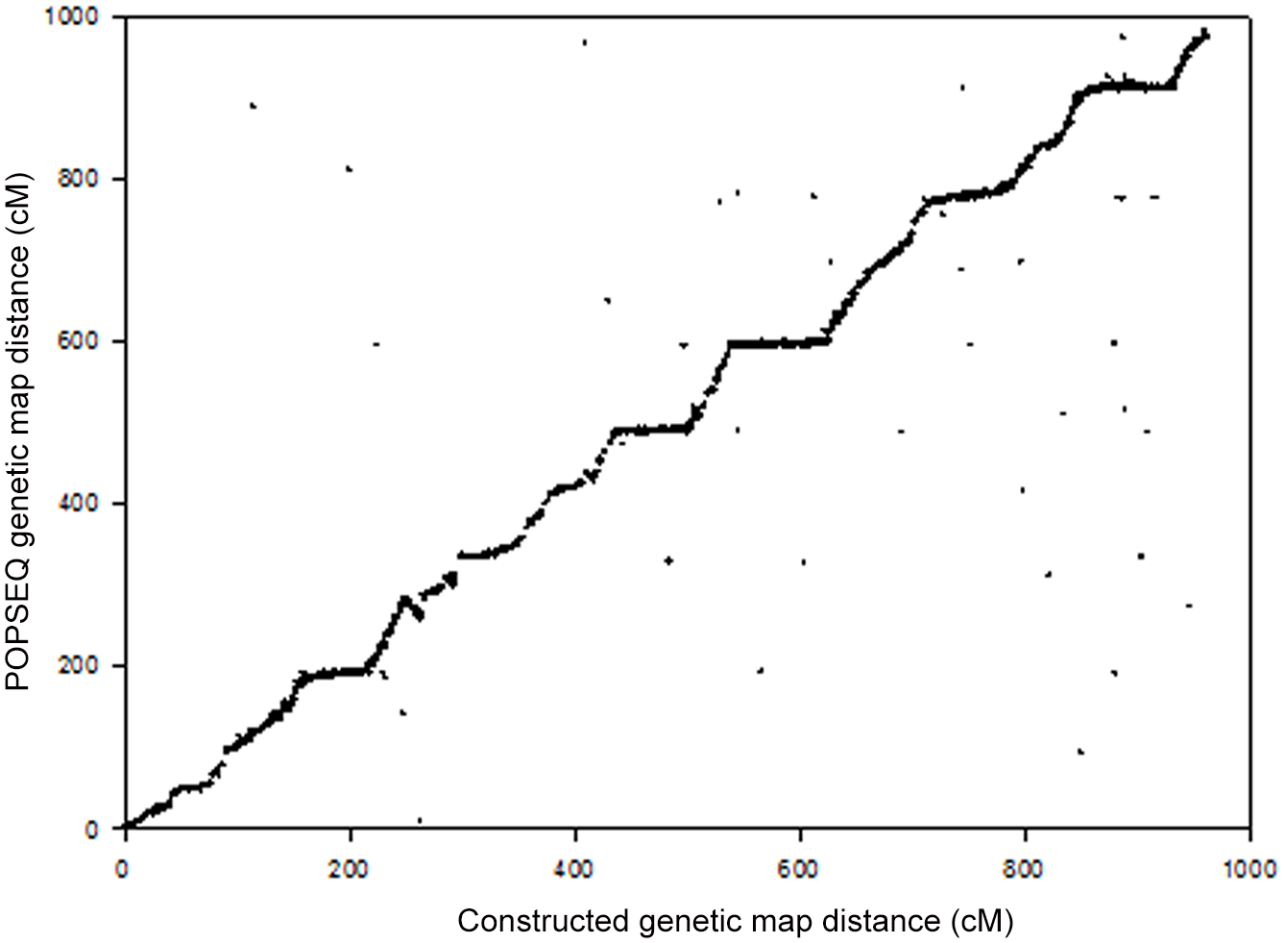
Alignment of genetic maps derived from de novo and POPSEQ. A total of 12,998 SNP markers were dotted in the figure. X-axis indicates the accumulative genetic position in the *de novo* genetic map; Y-axis indicates the accumulative genetic position in the POPSEQ genetic map.

The generated genetic map was aligned with the Morex/Barke reference genetic map which was used in the barley genomic DNA sequence assembly. Of the 12,998 markers, 91.4% were anchored to the barley reference genetic map (Morex/Barke). The genetic map constructed in this study was compared with the POPSEQ genetic map [6]. Only 35 SNP markers (0.3%) were located in different chromosomes (Fig 1). More than 99% of these SNP markers were located in the same chromosomes and genetic distances.

**Fig 2:**
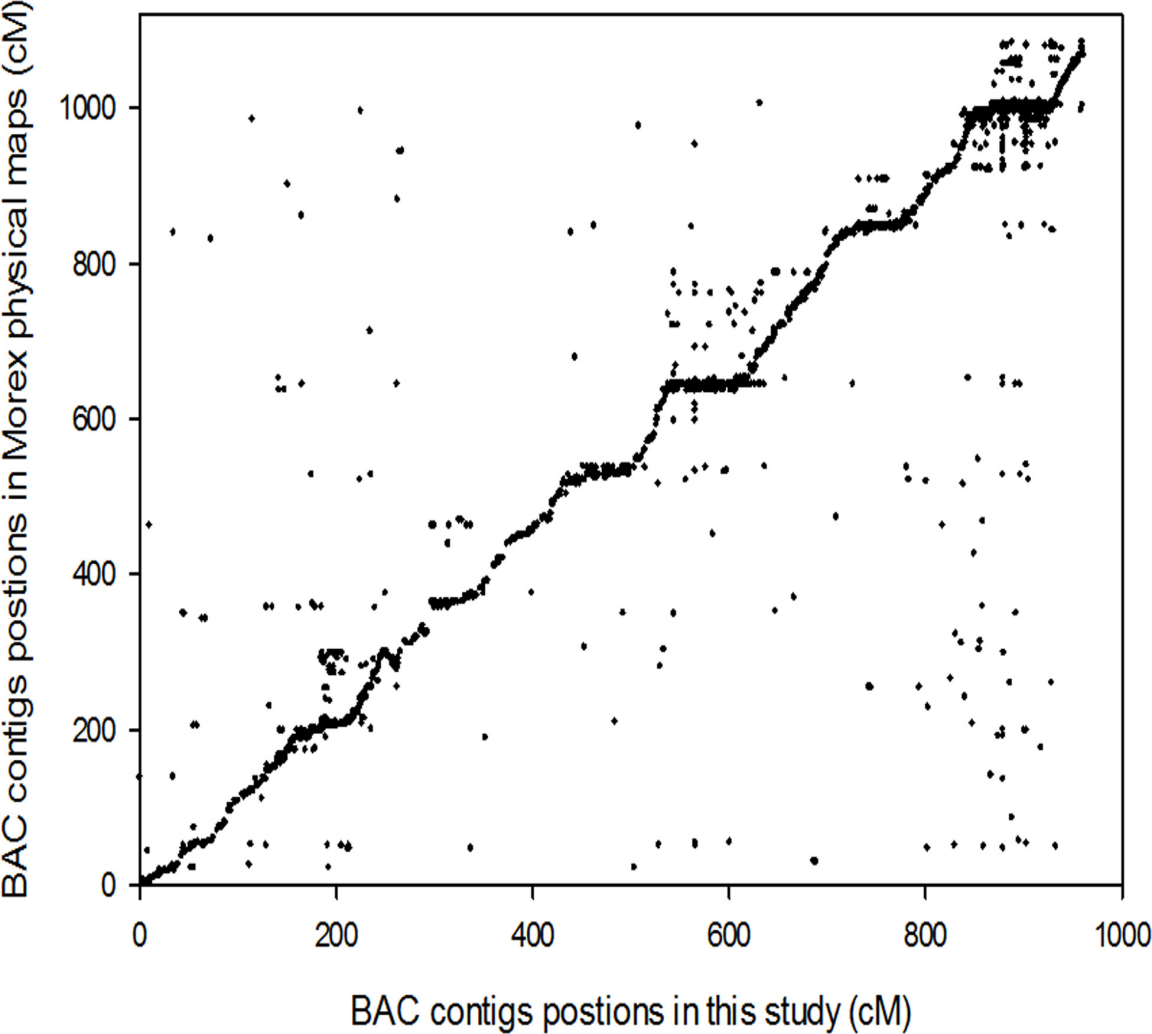
Alignment of BAC contig positions derived from de novo and POPSEQ. A total of 1,355 BAC contigs were dotted in the figure. X-axis indicates the accumulative genetic position in the *de novo* genetic map; Y-axis indicates the accumulative genetic position in the POPSEQ genetic map.

Furthermore, 4,095 SNP markers were anchored to 1,355 BAC contigs (Table 1). The BAC contig positions provided by this genetic map were compared with those provided by Mayer et al. [3]. About 1,227 BAC contigs (92.6%) were located in the same chromosomes in both genetic maps (Fig 2). The mismatched BAC contig positions can be corrected based on the high-density genetic map constructed by GBS.

### QTL mapping for average plant height

**Fig 3:**
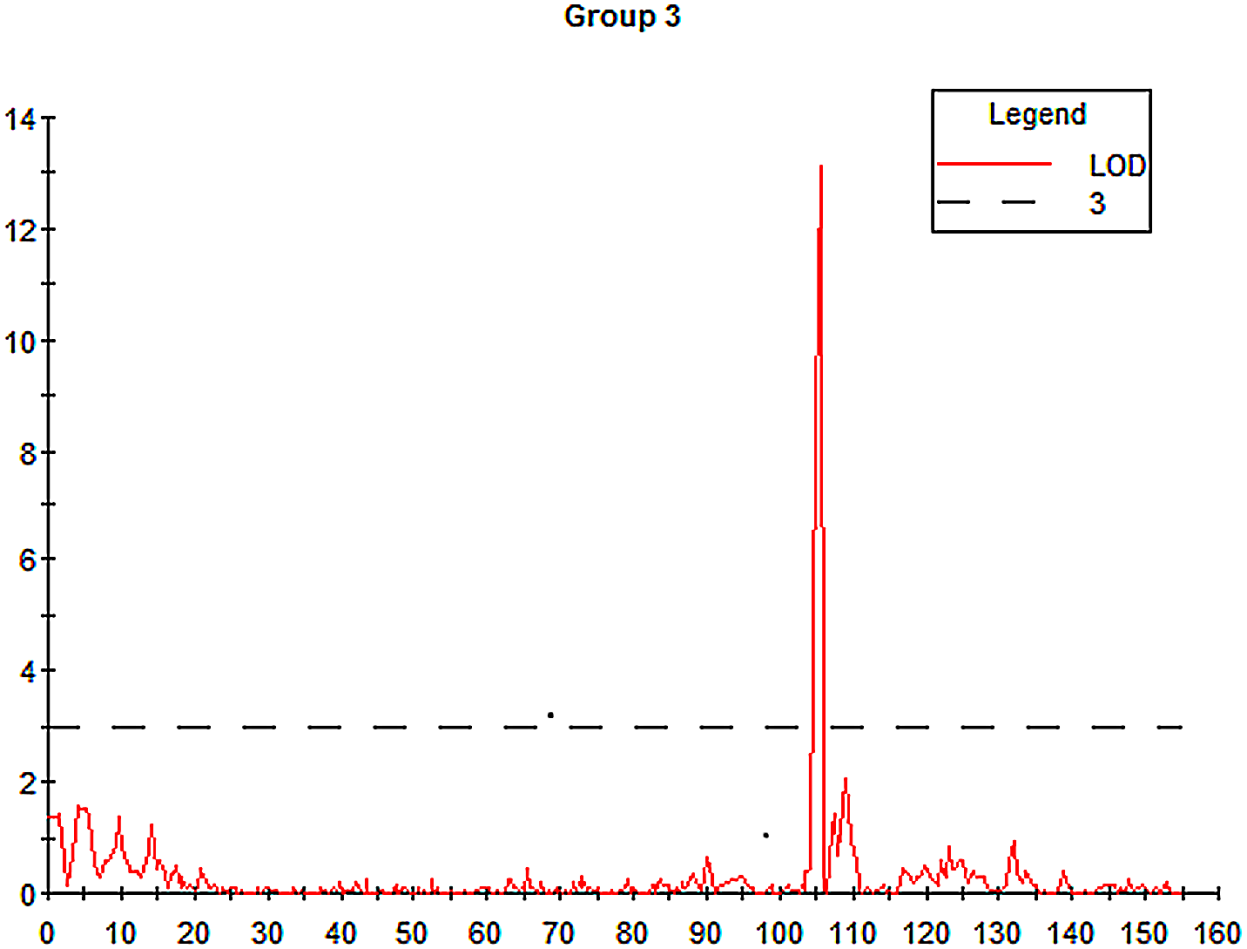
QTL mapping result for plant height in barley. Mapping QTL for plant height was conducted in the DH population of AC Metcalfe and Baudin in MapQTL5.0. In the chart, the red curve indicated LOD value at any genetic position. The black dash line indicated that the LOD value was 3.0.

Average plant heights for Baudin and AC Metcalfe were 61 cm and 81 cm, respectively. A single QTL for average plant height was identified on Chromosome 3H flanked with markers LG01_143803539 (104.9 cM) and LG01_25700534 (110.3 cM). There was a small peak with LOD value of ~2.0 in 109 cM region (Fig 3). Flanking markers covered all the putative genes surrounding these two peak regions. The precise region should be determined by recombinant lines. These two markers were anchored to shotgun sequence contigs of Morex_contig_50210 and Morex_contig_18277, respectively. Due to the limited BAC sequences, these two Morex contigs could not be linked to FPC. QTL analysis showed that the marker LG10_116465883 (105.5 cM) had the greatest LOD value of 13.01, and the marker’s additive effect was 8.9 cm (Fig 3). This locus explains 44.5% of the phenotypic variation.

## Discussion and Conclusions

NGS has been used in genome sequencing, such as wheat and barley [3,15]. When the cost is affordable for population sequencing, GBS is becoming more popular in genetic studies. For instance, Huang et al. [16] sequenced 150 rice recombinant lines and constructed a genetic map with resolution breakpoints within an average of 40 kb. The barley used in this study is from Canada (AC Metcalfe) and Australia (Baudin). These two varieties have been the international benchmark for malting quality for many years, and have been widely used as parents in many breeding programs. Although these two varieties have similar malting qualities, the genetic composition for malting quality differs [17]. They also differ in several agronomic traits and disease resistance, such as plant height, *P*. *neglectus* resistance and disease resistance [18]. In this study, we demonstrated that the high-density genetic map can be used effectively to map QTLs and identify underlying genes. The DH population derived from AC Metcalfe and Baudin together with the high-density genetic map will improve the accuracy of QTL mapping and facilitate fine mapping of these QTL, and eventually develop molecular markers for marker-assisted breeding.

Earlier generation genetic maps were constructed with around 3,000 SSR and DarT markers [19,20,21]. In this study, we identified and mapped around 13,000 SNP markers in one DH population. The density of molecular markers in this genetic map was about four times greater than earlier generation maps. A molecular map of the same population has been constructed using SSR and AFLP markers [19]. The total length of the map was 1306.6 cM with an average interval length of 5.6 cM between markers. In contrast, the density of markers in the present study was around 13 markers per cM. This approach is very robust and efficient to construct a *de novo* genetic map. It is worth mentioning that the map distance (967.6 cM) is about 25% shorter than the previous map (1,306.6 cM) which indicates that some early mapping data may need be rechecked.

The high-density genetic map constructed by GBS provides a framework for the assembly of WGS contigs. Previously, genetic maps and physical BAC contig maps were used to facilitate WGS contig assembly. For instance, Mayer et al. [3] anchored 308 Mb shotgun data directly to the sequence-enriched physical map by sequence homology. But construction of BAC libraries was time-consuming and costly. Later, they implemented a hierarchical method to further anchor the physical and genetic maps. A total of 4,556 BAC contigs totalling 3.9 Gb were assigned to chromosome arm bins based on a total of 3,241 SNP and 498,165 sequence-tag genetic markers, the majority in linear order. Finally, the WGS contigs were further validated and improved with high-density genetic maps derived from POPSEQ [6]. Overall, around 750,000 WGS contigs with a cumulative length of 1.222 Gb were anchored to two genetic maps using 11,229,709 SNPs [6]. In this study, about 7.4% of the BAC contigs were anchored to different positions when compared with Mayer et al. [3].

Poland et al. [5] only mapped 9,545 SNP markers with less than 20% missing rate to the OWB reference map. Increasing the missing rate to 80% placed 34,396 SNPs on the genetic map. Mascher et al. [6] constructed a *de novo* genetic map with around 60,000 SNPs with up to 17% missing rate. However, in our study, we constructed a *de novo* genetic map only using SNPs with less than 7% missing rate. The lower missing rate helps to improve the accuracy of the genetic map.

Plant height was mapped at 105.5 cM on chromosome 3H. Whole-genome gene annotations can be downloaded from ftp://ftp.ipk-gatersleben.de. One gene gibberellin 20-oxidase-2 (MLOC_56462.1, 107.9 cM) was 2.4 cM away from the QTL marker LG10_116465883 (105.5 cM). QTL mapping result was consistent with the report described by Jia et al. [22], in which a single QTL was identified and mapped to chromosome 3H region as well. They also illustrated that the expression level of the gene *Hv20ox*_*2*_ within the QTL region was associated with plant height [22]. In this study, in order to fine-map the QTL region, a larger population should be used to screen recombinant lines. By combining genotypes and phenotypes of these recombinant lines, the QTL region should be narrowed down. Therefore, whether plant height is encoded by the gene gibberellin 20-oxidase-2 needs to be further studied. This study indicates that combining high-density genetic maps with gene annotations will facilitate map-based cloning.

Some SNP markers can be converted to InDel (insert/deletion) markers based on a nearby InDel locus. SNP marker tag sequences were used to blast barley genomic sequences of Morex, Barke and Bowman. By aligning these sequences, a new InDel marker can be developed in the InDel region. These InDel markers can be scored in 1% agarose gel or PAGE gel instead of using special instruments. New markers within the QTL region can also be developed as described above for map-based cloning.

In conclusion, we have described an approach to construct a high-density genetic map through GBS which will facilitate QTL mapping and fine mapping. The linear SNP marker sequences can be used to improve physical map construction. Furthermore, this approach can be applied to other species where recombinant lines can be obtained.

## Supporting Information

S1 File The details of SNP markers. The file includes the SNP markers names, NCBI submitted SNP (ss) accession numbers, genetic positions in this map and POPSEQ map, tag sequences, anchored Morex contig and individual BAC, and anchored BAC contig. N/A means that the marker tag cannot be anchored to the database.
(XLSX)
